# The development of visual aesthetic sensitivity in students in China

**DOI:** 10.3389/fpsyg.2023.1071487

**Published:** 2023-02-28

**Authors:** Ju Zhang, Xu Du, XiaoWei Zhang, XueJun Bai

**Affiliations:** ^1^Faculty of Psychology, Tianjin Normal University, Tianjin, China; ^2^Department of Applied Psychology, Law School, Southwest University of Science and Technology, Mianyang, China; ^3^Zunyi Normal University, Zunyi, China

**Keywords:** development, visual aesthetic sensitivity, students, artistic training, gender difference

## Abstract

To examine the development of visual aesthetic sensitivity in students in China, 2,387 students from age 9 to age 22 (excluding ages 16–17) were tested by the Visual aesthetic Sensitivity Test-Revised. The development of visual aesthetic sensitivity across ages and genders, and the effect of artistic training on students’ visual aesthetic sensitivity were examined. The data of primary school and junior middle school students were collected by paper tests completed collectively in class, while the data of university students were collected by distributing and collecting online. Result suggests that students’ visual aesthetic sensitivity is relatively stable from age 9 to age 12 and increases at age 13. The visual aesthetic sensitivity of girls is significantly better than that of boys at age 15, 19, and 20 years of age. This study also found that artistic training improves students’ visual aesthetic sensitivity.

## 1. Introduction

Aesthetic sensitivity is a notion to distinguish individuals’ differences in aesthetic ability (Myszkowski and Zenasni, 2016; Myszkowski et al., 2018; Myszkowski, 2020). Researchers suggest that individuals may differ in aesthetic sensitivity which affects their aesthetic judgments (Myszkowski and Zenasni, 2016; Myszkowski et al., 2018). Aesthetic sensitivity is of great importance for creative people, especially those engaged in art (Duffy, 1979a,b). Improving visual aesthetic sensitivity can enhance an individual’s aesthetic ability and aesthetic taste, which have a positive impact on people’s mental health (Myszkowski, 2020). There are few studies on the natural development of aesthetic sensitivity, but this field requires more research (Myszkowski, 2020). The current study examines the development of aesthetic sensitivity in students in China, from age 9 to age 22 (excluding ages 16–17), and the effect of artistic training on students’ visual aesthetic sensitivity.

### 1.1. Aesthetic sensitivity

Aesthetic *sensitivity* refers to the degree to which a person responds to relevant aesthetic stimuli in a way that is consistent and appropriate with external aesthetic value standards (Child, 1964). This is the clearest definition with more details and less controversial (Myszkowski, 2020). Aesthetic sensitivity is regarded as the scientific terminology for Eysenck’s “good taste” (Eysenck, 1940), which can reflect the difference in individual aesthetic ability (Götz et al., 1979; Marschallek et al., 2019; Myszkowski, 2019), mainly reflected in the identification of aesthetic quality and judging the quality of artworks (Duffy, 1979b; Myszkowski, 2020).

### 1.2. Aesthetic sensitivity and cognitive ability

The concept of aesthetic sensitivity is closely related to aesthetic experience (Clemente, 2022). Aesthetic sensitivity is the ability to identify the difference between harmonious and excellent design (Eysenck et al., 1984; Myszkowski et al., 2014). “Aesthetic sensitivity was introduced as a way to account for a form of intelligence that was not (enough) represented in typical cognitive ability testing” (Myszkowski, 2020, p. 9). Researchers have found that aesthetic sensitivity is related to general intelligence and personality (Myszkowski et al., 2014, 2018; Myszkowski, 2020). The relation between visual aesthetic sensitivity and intelligence does not significantly changed by age and gender, and they shared some cognitive processes (Myszkowski et al., 2018; Myszkowski, 2020). Aesthetic sensitivity is also related to divergent thinking ability, especially figural divergent thinking ability, which is very important to creativity (Myszkowski et al., 2014). It is significantly correlated with preference for complexity, artistic potential, artistic interest, extroversion and field independence (Duffy, 1979a,b; Summerfeldt et al., 2015).

### 1.3. The development of visual aesthetic sensitivity

Some studies posit that visual aesthetic sensitivity increases with age or educational grade, and there are gender differences in some age groups. Duffy (1979a) viewed aesthetic sensitivity to accelerate approximately 11–12 years old, after which a constant development period until 14–15 years old appears, and there is a period of acceleration and high development that lasts until young adulthood. Eysenck (1972b) found that the visual aesthetic sensitivity scores of junior school students in all grades are higher than those of primary school students, those of art majors are higher than those of primary and junior school students, and non-art majors. Duffy (1979b) suggested that visual aesthetic sensitivity scores are stable between grades 4–6 but increase from grades 8–10. Frois and Eysenck (1995) suggested that visual aesthetic sensitivity increase at 10–14 years old and that girls’ sensitivity at 11-, 12- and 13 years old is significantly higher than that of boys. Some studies have also shown that visual aesthetic sensitivity is not related to age or gender. Götz et al. (1979) suggested that there is no significant difference in visual aesthetic sensitivity based on age and gender. Two other related studies found similar results regarding age (Iwawaki et al., 1979; Chan et al., 1980). The results on the development of visual aesthetic sensitivity by age/grade and gender are inconsistent. The differences in test materials may lead to difference results (Eysenck, 1972a), so it is impossible to draw a consistent conclusion. Several studies used different test materials of visual aesthetic sensitivity, such as the Maitland Graves Design Judgment Test (Eysenck, 1972b), the Child’s Aesthetic Sensitivity Test (Duffy, 1979b) and the VAST (50-item final edition; Frois and Eysenck, 1995), while others used the same test materials but different versions, such as Frois and Eysenck using the VAST (50-item final edition; 1995); however, Götz et al. (1979), Iwawaki et al. (1979), and Chan et al. (1980) used the VAST (42-item version).

### 1.4. Artistic training on visual aesthetic sensitivity

The findings on whether artistic training affects the development of visual aesthetic sensitivity vary across studies. Artistic training in this paper means formal education in visual art. Myszkowski (2020) suggested that art expertise gained through formal education improves one’s visual aesthetic sensitivity. Duffy (1979b) claimed that by developing children’s perceptual discrimination ability, critical thinking and learning vocabulary related to emotion and aesthetic quality in operation stage, most children’s aesthetic sensitivity will increase. The artistic expertise acquired through education or training can increase the degree to which artistic works are liked (van Paasschen et al., 2015). People with artistic training spend more time on the overall recognition of the image structure of the background features of artistic works and their relationship with narrative themes (Nodine et al., 1993). There are also studies that show the development of visual aesthetic sensitivity to be unrelated to artistic training (Eysenck, 1972b; Frois and Eysenck, 1995) but only with individual maturity (Götz et al., 1979).

### 1.5. Visual aesthetic sensitivity test and its revision

The Visual Aesthetic Sensitivity Test (VAST) was compiled by Götz et al. (1979) and revised by Götz (1985). It is widely used to examine visual aesthetic sensitivity (Chan et al., 1980; Eysenck, 1983; Frois and Eysenck, 1995; Myszkowski et al., 2014; Myszkowski and Storme, 2017; Mitrovic et al., 2020; Myszkowski, 2020; Myszkowski and Zenasni, 2020). There are other historical aesthetic sensitivity tests, such as the Maitland Graves Design Judgment Test (Graves, 1948) and the Meier Art Tests (Meier, 1940). The only way for these tests to make content validity effective was to use the “controlled alteration” approach (Meier, 1928; Seashore, 1929; Myszkowski et al., 2018; Myszkowski, 2020). However, for the VAST, the content validity is also verified by consensus of community samples and expert validity (Myszkowski and Storme, 2017). The VAST has good psychometric characteristics (Stich, 2005), making it the only psychometric test of aesthetic sensitivity that is worth recommending (Myszkowski and Storme, 2017). There are 50 items in the VAST, and each item consists of a pair of nonrepresentational pictures present side by side for choice: one is the originals, and the other is altered, which is weaker in aesthetic quality (Götz, 1985). Based on this aesthetic standard (Tepe, 2021), if participants choose the original picture, they receive 1 point; otherwise, they receive 0 point. The test score ranges from 0 to 50.

Although researchers have studied the internal consistency and the external criterion validity of the VAST (Götz et al., 1979; Chan et al., 1980; Eysenck et al., 1984; Frois and Eysenck, 1995; Myszkowski et al., 2014; Myszkowski and Storme, 2017), no researchers have studied its structural validity. Myszkowski and Storme (2017) examined and revised the structural validity of the VAST to obtain revised version (VAST-R). The VAST-R retained 25 items and had satisfactory internal consistency and structural validity (Myszkowski and Storme, 2017).

### 1.6. The current study

Overall, some progress has been made in the research on the development of visual aesthetic sensitivity, but there are some inconsistencies: whether visual aesthetic sensitivity increases with age, whether there are gender differences, and whether it is affected by artistic training. The current study uses the VAST-R to examine students’ development of aesthetic sensitivity from age 9 to age 22 (excluding ages 16–17) and the influence of artistic training in China. Based on the research available, we have two hypotheses. First, we assume that students’ visual aesthetic sensitivity increases with age and that there are significant gender differences at some age levels. Second, we assume that artistic training improves students’ visual aesthetic sensitivity.

## 2. Materials and methods

### 2.1. Participants

Participants were recruited from 4 primary schools, 5 junior schools and 2 universities in Tianjin and Mianyang. Primary schools and junior schools are located in urban and rural areas. In China, students in grades one to six go to primary school (usually aged 6 to 12), and students in grades 7–9 go to secondary school (usually aged 13 to 15). Students usually go to university at approximately 18 years old. A total of 1,100 paper tests were distributed in primary and junior schools, and 1,088 copies were recovered. The tests were entered into electronic documents, 41 invalid tests (missing one question or more) were eliminated, and 1,047 valid tests were obtained. A total of 1,402 data points from university students were obtained, with *M* = 381.50 s and SD = 238.77 s, excluding 62 data points exceeding one standard deviation lower than the average, and 1,340 valid tests were obtained, accounting for 4.42% of the university data. The number of participants by age and gender is shown in Table 1. All the participants were physically and mentally healthy, with normal or corrected vision, and had never participated in similar research.

**Table 1:** Number, major of participants by age and gender.

Age	Majors	*n*	Boys	Girls
8	2	16	5	11
9	2	88	44	44
10	2	116	58	53
11	2	109	47	62
12	2	161	65	96
13	2	247	127	120
14	2	200	98	102
15	2	105	59	46
16	2	5	3	2
17	2	7	0	7
18	1	35	11	24
	2	148	73	75
19	1	75	28	47
	2	267	171	96
20	1	86	35	51
	2	384	247	147
21	1	31	14	17
	2	225	120	105
22	1	10	8	2
	2	56	29	27
23	2	9	2	7
24	1	1	1	0
	2	6	6	0
Total	1	238	97	141
	2	2,149	1,159	990

Major: 1 = art-related majors, 2 = non-art-related majors.

### 2.2. Procedures and materials

The data of primary and junior middle school participants were collected by paper tests completed collectively in class, while the data of university participants were collected by distributing and collecting online.

The participants were asked about their grade, age, and gender. University students were also asked about their majors. Students majoring in art design, environmental art design and other visual arts related majors were regarded as art-related majors, while other majors such as Chinese language and literature, law and computer science were regarded as non-art-related majors. All the participants then completed the VAST-R. There was no time limit for the test.

## 3. Results

### 3.1. Verify the degree of fitness between the valid data and the VAST-R

To verify the degree of fitness between the valid data and the VAST-R, confirmatory factor analysis was carried out on all valid data. The software used was Mplus 8.8, and *χ^2^/df* = 3.043, *RMSEA* = 0.029, *CFI* = 0.984, *TLI* = 0.974, *SRMR* = 0.016. The internal consistency Cronbach’s *α* of all valid VAST-R data is 0.631.

### 3.2. Development of visual aesthetic sensitivity

#### 3.2.1. Age and gender differences in visual aesthetic sensitivity

To assess differences in aesthetic sensitivity across ages and genders, all valid data (except art-related majors) were included in the analysis, and the internal consistency Cronbach’s *α* of the VAST-R was.622. The number of participants by age and gender is shown in Table 1. Because of the small number of age 8 participants, they were merged into the age 9 group. For similar reasons, age 16 was merged into the age 15 group, age 17 was merged into age 18, and ages 23–24 were merged into the age 22 group. For university students, only non-art-related majors were included in the analyses to avoid possible interference caused by artistic training. The number, major, mean ages, and standard deviations of university participants by grade and gender are shown in Table 2.

**Table 2:** Number, major, mean ages, and standards deviations of university participants by grade and gender.

Grade	Total			Boys		Girl	
	Major	*n*	*M_age_*	*n*	*M_age_*	*n*	*M_age_*
13	1	99	18.94 (0.79)	33	19.09 (0.88)	66	18.86 (0.74)
	2	322	18.68 (0.76)	183	18.76 (0.77)	139	18.56 (0.73)
14	1	84	19.68 (0.75)	44	19.80 (0.85)	40	19.55 (0.60)
	2	436	19.88 (0.84)	328	19.96 (0.85)	108	19.65 (0.77)
15	1	55	20.76 (0.88)	20	21.15 (1.09)	35	20.54 (0.66)
	2	344	20.82 (0.86)	140	20.87 (0.87)	204	20.78 (0.86)
Total	1	238	19.62 (1.06)	97	19.83 (1.17)	141	19.48 (0.96)
	2	1,102	19.82 (1.17)	651	19.82 (1.12)	451	19.82 (1.24)

Major: 1 = art-related majors, 2 = non-art-related majors.

The VAST-R scores were analyzed by a 12 (age level: 9, 10, 11, 12, 13, 14, 15, 18, 19, 20, 21, 22) × 2 (gender: male, female) two-factor ANOVA, and all the factors were between subjects. *Post hoc* tests were conducted to examine how aesthetic sensitivity varied by age level and gender.

**Figure 1:**
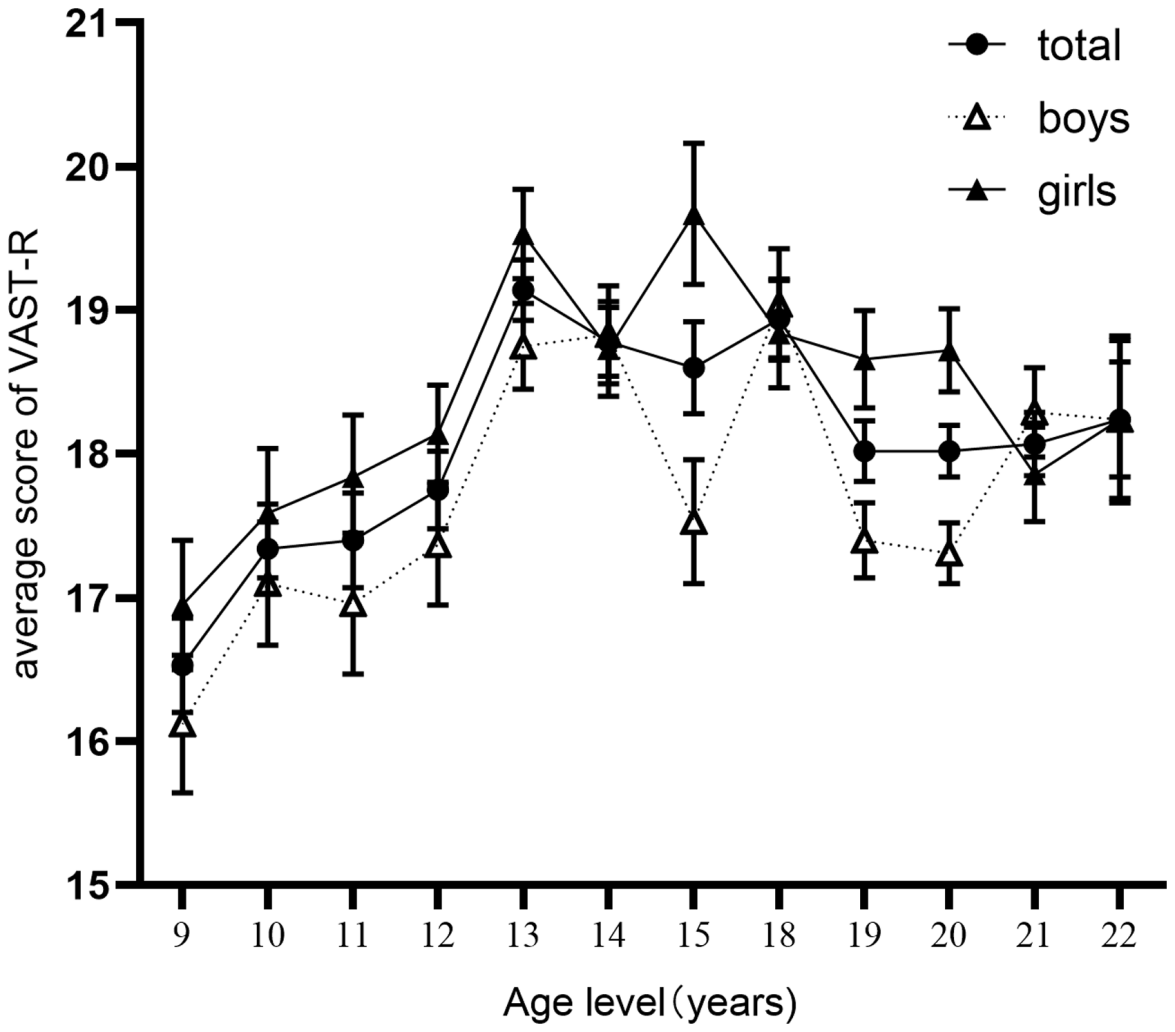
Mean and standard error of VAST-R scores of total and gender by age level.

The main effect of age was significant [*F*(11, 2,125) = 7.10, *p*< 0.001, $\eta_{p}^{2}$ = 0.035]. See Table 3 for means and standard deviations of VAST-R scores by age and gender. Follow-up *post hoc* pairwise analyses (Figure 1) showed that for VAST-R scores, students at age 9 scored significantly lower than those at ages 13, 14, 15, 18, 20, and 21. Students at age 10 scored significantly lower than those at ages 13, 14, and 18. Students at age 11 scored significantly lower than those at ages 13 and 18. Students at age 12 scored significantly lower than those at age 13. Students at age 13 scored significantly higher than those at ages 19 and 20. Students aged 18 scored significantly higher than those aged 20. There were no other significant differences in VAST-R scores by age. In general, visual aesthetic sensitivity was relatively stable from age 9 to age 12, and it increased at age 13. After that, visual aesthetic sensitivity was relatively stable again.

**Table 3:** Means (standard deviations) of VAST-R scores by age and gender.

Age	Total		Boys		Girls	
	*n*	*M*	*n*	*M*	*n*	*M*
9	104	16.56 (3.24)	49	16.12 (3.21)	55	16.95 (3.24)
10	116	17.34 (3.30)	60	17.10 (3.33)	56	17.59 (3.27)
11	109	17.46 (3.35)	47	16.96 (3.36)	62	17.84 (3.33)
12	161	17.83 (3.20)	65	17.37 (3.50)	96	18.14 (2.96)
13	247	19.13 (2.79)	98	18.83 (2.83)	102	18.73 (3.65)
14	200	18.78 (3.26)	98	18.83 (2.83)	102	18.73 (3.65)
15	110	18.46 (4.00)	62	17.53 (4.14)	48	19.67 (3.50)
18	155	18.94 (3.17)	76	19.04 (2.90)	79	18.84 (3.42)
19	267	17.85 (3.61)	171	17.40 (3.75)	96	18.66 (3.21)
20	384	17.82 (3.68)	247	17.32 (3.68)	137	18.72 (3.49)
21	225	18.09 (3.40)	120	18.29 (3.34)	105	17.86 (3.46)
22	71	18.24 (3.24)	37	18.24 (3.45)	34	18.24 (3.04)

The main effect of gender was also significant [*F*(1, 2,125) = 15.96, *p*< 0.001, $\eta_{p}^{2}$ = 0.007]. Follow-up *post hoc* pairwise analyses showed that for VAST-R scores, the scores of girls (*M* = 18.48, SD = 3.32) were significantly higher than those of boys (*M* = 17.79, SD = 3.50; *p* < 0.001).

There was a significant interaction between age and gender [*F*(11, 2,125) = 2.14, *p* = 0.015, $\eta_{p}^{2}$ = 0.011]. Further simple effect analysis showed that with boys (Figure 1), the 9-year-olds scored significantly lower than the 12, 13, 14, 15, 18, 19, 20, 21, and 22-year-olds. The 10-year-olds scored significantly lower the 13, 14, 18, and 21-year-olds. The 11-year-olds scored significantly lower than the 13, 14, 18, and 21-year-olds. The 12-year-olds scored significantly lower than the 13, 14, and 18-year-olds. The 13-year-olds scored significantly higher than the 15, 19, and 20-year-olds. The 14-year-olds scored significantly higher than the 15, 19, and 20-year-olds. The 15-year-olds scored significantly lower than the 18-year-olds. The 18-year-olds scored significantly higher than the 19 and 20-year-olds. The 19-year-olds scored significantly lower than the 21-year-olds. The 20-year-olds scored significantly lower than the 21-year-olds. There were no other significant age level differences for boys. These results showed that boys’ visual aesthetic sensitivity was relatively stable from age 9 to age 12 and significantly lower than that at ages 13, 14- and 18-year-olds. From age 13, the visual aesthetic sensitivity of boys improves and is similar to that of 14 and 18 years old. In addition, there is no regular change.

Further simple effect analysis showed that with girls (Figure 1), the 9-year-olds scored significantly lower than the 12, 13, 14, 15, 18, 19, and 20-year-olds. The 10-year-olds scored significantly lower than the 13, 14, 15, 18, and 20-year-olds. The 11-year-olds scored significantly lower than the 13 and 15-year-olds. The 12-year-olds scored significantly lower than the 13 and 15-year-olds. The 13-year-olds scored significantly higher than the 21 and 22-year-olds. The 15-year-olds scored significantly higher than the 21-year-olds. The 20-year-olds scored significantly higher than the 21-year-olds. There were no other significant age level differences for girls. Similarly, these results showed that girls’ visual aesthetic sensitivity is relatively stable from age 9 to age 12 and significantly lower ages 13 and 15. From age 13, the visual aesthetic sensitivity of girls improves, and is similar to that of 15-year-olds. Apart from this, there is no regular change in visual aesthetic sensitivity.

Further simple effect analysis shows that for age, the VAST-R scores of girls were significantly higher than those of boys at age 15 [*F*(1, 2,125) = 10.93, *p* < 0.001, $\eta_{p}^{2}$ = 0.005], age 19 [*F*(1, 2,125) = 8.64, *p* = 0.003, $\eta_{p}^{2}$ = 0.004] and age 20 [*F*(1, 2,125) = 15.31, *p* < 0.001, $\eta_{p}^{2}$ = 0.007]. There were no other significant differences.

### 3.3. The effect of artistic training on visual aesthetic sensitivity

To assess the effect of artistic training on visual aesthetic sensitivity, the VAST-R scores were analyzed by a 3 (grade: 13, 14, 15) × 2 (artistic training: art-related majors, non-art-related majors) two-factor ANOVA, and all the factors were between subjects. *Post hoc* tests were conducted to examine how aesthetic sensitivity varied by grade level and gender. Art-related majors were regarded as students who had formal artistic training in visual art, while non-art-related majors were regarded as students who did not have formal artistic training in the visual art (Eysenck, 1972a; Frois and Eysenck, 1995; Francuz et al., 2018). The number, major, mean ages, and standard deviations of university participants by grade and gender are shown in Table 2.

The main effect of grade was significant [*F*(1, 1,334) = 8.91, *p* = 0.032, $\eta_{p}^{2}$ = 0.005]. The means and standard deviations (standard deviation in brackets) of the VAST-R scores of the three grades were as follows: 18.60 (3.25), 17.71 (3.85), and 18.73 (3.30). Follow-up *post hoc* pairwise analyses showed that the VAST-R scores of the freshmen were significantly higher than those of the sophomores (*p* < 0.001), with an average difference of 0.89, but the average difference between the freshmen and juniors was not significant. The sophomores scored significantly lower than the juniors (*p* < 0.001), with an the average difference of 1.03.

The main effect of artistic training was significant [*F*(1, 1,334) = 28.28, *p* < 0.001, $\eta_{p}^{2}$ = 0.021]. For the art-related majors, the means and standard deviations (standard deviation in brackets) from the freshman to junior were as follows: 18.37 (3.29), 19.98 (3.29), and 20.13 (3.47). For the non-art-related majors, the means and standard deviations (standard deviation in brackets) from freshman to junior were as follows: 18.67 (3.24), 17.27 (3.80), and 18.51 (3.23). Follow-up *post hoc* pairwise analyses showed that for the VAST-R scores, the scores of art-related majors (*M* = 19.34, SD = 3.42) were significantly higher than those of non-art-related majors (*M* = 18.06, SD = 3.52; *p* < 0.001).

There was a significant interaction between grade and artistic training [*F*(2, 1,334) = 14.19, *p* < 0.001, $\eta_{p}^{2}$ = 0.021]. Further simple effect analysis showed that for the art-related majors, the freshmen scored significantly lower than the sophomores (*p* = 0.002), with an average difference of 1.60, and they also scored significantly lower than the juniors (*p* = 0.003), with an average difference of 1.75. The average difference between the sophomores and juniors was not significant. For the non-art-related majors, the freshmen scored significantly higher than sophomores (*p* < 0.001); the average difference was 1.40, and the average difference between the sophomores and juniors was not significant. The sophomores scored significantly lower than the juniors (*p* < 0.001), and the average difference was 1.24. Further simple effect analysis showed that for freshmen, the VAST-R scores difference between the art-related majors and non-art-related majors was not significant. For the sophomores, the art-related majors scored significantly higher than the non-art-related majors (*p* < 0.001), with an average difference of 2.71. For the juniors, the art-related majors also scored significantly higher than the non-art-related majors (*p* = 0.001), and the average difference was 1.62.

These results showed that the visual aesthetic sensitivity of the freshmen was similar, but that of the art-related majors was increased and significantly higher than that of the non-art-related major sophomores and juniors, indicated that artistic training improved students’ visual aesthetic sensitivity.

## 4. Discussion

In this study, the VAST-R was used to examine the development of students’ visual aesthetic sensitivity, gender differences and the effect of artistic training from age 9 to age 22 (excluding ages 16–17).

This study found that visual aesthetic sensitivity increases with age, which is consistent with previous studies that also found visual aesthetic sensitivity increases with age (Duffy, 1979a,b; Frois and Eysenck, 1995), but this increase only occurs at the age of 13 and remains stable before that, which is different from previous studies. The visual aesthetic sensitivity of girls at the ages of 15, 19, and 20 is significantly higher than that of boys, which is a new departure from previous research. This study also found that art training is helpful in improving the visual aesthetic sensitivity. These findings show that visual aesthetic sensitivity increases with age, and this growth will only occur at a certain age, rather than increasing year by year within a certain age range. The developmental process for visual aesthetic sensitivity is not completely consistent between boys and girls. Attention should be given to the role of aesthetic training in improving visual aesthetic sensitivity.

### 4.1. Visual aesthetic sensitivity increases at age 13

This research found that visual aesthetic sensitivity is relatively stable in primary school children from age 9 to age 12 and increases at age 13. These results are consistent with the findings of Frois and Eysenck (1995) and Eysenck (1972b). This finding is also partly consistent with the findings of Duffy’s research (Duffy, 1979a). The increase in students’ visual aesthetic sensitivity in junior school may be related to the improvement of students’ understanding and tolerance for the emotional quality of pictures (Child, 1971; Duffy, 1979b). Students’ understanding of painting varies across stages of cognitive development (Vivaldi et al., 2020). Based on Piaget’s cognitive development theory (Lehalle, 2020), the significant changes in aesthetic judgment may be related to the change in students’ thinking mode from concrete-operational stage to the formal-operational stage, and the cognitive strategies have also changed accordingly. Aesthetic development is related to but not determined by age, and aesthetic experience plays an important role in aesthetic development; adult are not in a higher aesthetic stage just because they are older (DeSantis and Housen, 2000). This could be a reason for the visual aesthetic sensitivities of the junior school and university students were similar.

### 4.2. There are gender differences in students’ visual aesthetic sensitivity

This research found that the visual aesthetic sensitivity of boys and girls is relatively stable from age 9 to age 12 and increases at age 13. This shows that the development processes of boys and girls are synchronous at 9–13 years old. However, the visual aesthetic sensitivity of girls is significantly higher than that of boys at 15, 19, and 20 years of age, which is consistent with the assumption of this study. Myszkowski (2019) suggested that the differences in individual visual aesthetic sensitivity are related to the extent to which individuals extensively process aesthetic objects. Visual aesthetic sensitivity is related to divergent thinking ability (Myszkowski et al., 2014). Therefore, the gender differences in visual aesthetic sensitivity may be related to the difference of divergent thinking or creativity between male and female students. Researchers showed that boys and girls differed across scales of divergent thinking (Lau and Cheung, 2010). Men generally show greater differences in the overall distribution of creativity scores in divergent thinking, and there is greater variability in graphic creativity among males (He and Wong, 2021). Bart et al. (2015) showed that there are significant differences between males and females in most creative thinking subtests among students in Grade 8 and Grade 11, and females have obvious advantages.

### 4.3. Artistic training improves visual aesthetic sensitivity

This study found that the overall aesthetic sensitivity of art-related majors is significantly higher than that of non-art-related majors. Students with formal artistic training have better visual aesthetic sensitivity than those without artistic training, which is consistent with Duffy’s (1979b) viewpoint, indicating that artistic training can improve students’ visual aesthetic sensitivity. Bimler et al. (2019) suggested that experts can use more complicated strategies to analyze in art appreciation. Duffy (1979a) viewed that in the specific operation stage, visual aesthetic sensitivity can be improved by guiding children to develop their cognitive abilities, critical thinking and to learn more vocabulary. Hekkert and van Wieringen (1996) suggested that the different aesthetic evaluations of original and altered pictures by viewers with different expertise levels are related to artistic training.

### 4.4. Limitations and future directions

This study has some limitations. First, the number of participants in each age group was not balanced. We will pay attention to this in future research. Second, our research lacks the test data of high school students aged 16–17, and the data could be increased in the future. In addition, this study is a cross-sectional study, preventing conclusions about the development of visual aesthetic sensitivity in the same group of individuals from being drawn (Barbot, 2019; Zyga et al., 2022).

## 5. Conclusion

The current study examined the development of visual aesthetic sensitivity, gender differences, and the effect of artistic training on students in China by using the VAST-R. This result suggest that students’ visual aesthetic sensitivity is relatively stable from age 9 to age 12 and increases at age 13. The visual aesthetic sensitivity of girls is significantly better than that of boys at 15, 19, and 20 years of age. This study also found that artistic training improved students’ visual aesthetic sensitivity.

Our research has addressed the lack of mainland Chinese participants in previous studies on visual aesthetic sensitivity. The results support previous findings that visual aesthetic sensitivity increases with age and indicate this growth will only occur at a certain age, rather than increasing year by year within a certain age range. The developmental process for visual aesthetic sensitivity is not completely consistent between boys and girls. Art training helps improve visual aesthetic sensitivity, which increases the confidence of aesthetic researchers and all people who want to improve visual aesthetic sensitivity.

## Data availability statement

The raw data supporting the conclusions of this article will be made available by the authors, without undue reservation.

## Ethics statement

The studies involving human participants were reviewed and approved by the Research Ethics Committee of Tianjin Normal University. Written informed consent to participate in this study was provided by the participants’ legal guardian/next of kin.

## Author contributions

JZ conceived of the study, participated in its design, performed the measurement, coordinated, and drafted the manuscript. XD aided in data analyses and revised the manuscript. XZ participated in the design and collected the data. XB provided guidance on the development of the project, assisted in the study design, and revised the manuscript. All authors contributed to the article and approved the submitted version.

## Conflict of interest

The authors declare that the research was conducted in the absence of any commercial or financial relationships that could be construed as a potential conflict of interest.

## Publisher’s note

All claims expressed in this article are solely those of the authors and do not necessarily represent those of their affiliated organizations, or those of the publisher, the editors and the reviewers. Any product that may be evaluated in this article, or claim that may be made by its manufacturer, is not guaranteed or endorsed by the publisher.
